# Short-Term Efficacy of Endoscopic Sleeve Gastroplasty Versus Intragastric Balloon Insertion for Obesity: An Indonesian, Single-Center, Retrospective Cohort Study

**DOI:** 10.7759/cureus.67355

**Published:** 2024-08-20

**Authors:** Peter I Limas, Jeffrey Budhipramono, Andre S Suryadi, Adrian P Setiawan, Lady D Alfara

**Affiliations:** 1 Gastrointestinal Surgery, Sumber Waras Hospital/Tarumanegara University, Jakarta, IDN; 2 Surgery, Sumber Waras Hospital/Tarumanegara University, Jakarta, IDN; 3 Medicine, Sumber Waras Hospital, Jakarta, IDN; 4 Physician Nutrition; Metabolic Disorders Nutrition, Sumber Waras Hospital, Jakarta, IDN

**Keywords:** weight loss, obesity, gastroplasty, efficacy, balloon insertion

## Abstract

Introduction

As a medical condition, obesity is a global public health concern that still has no satisfactory solution. Endoscopic sleeve gastroplasty (ESG) and intragastric balloon (IGB) are proven to be safe and efficient in producing weight loss. Endoscopic sleeve gastroplasty has achieved significant success; therefore, it is timely to compare it to intragastric balloon therapy.

Methods

We retrospectively reviewed prospectively collected data for patients undergoing ESG or IGB. Weight was recorded at one week, one month, and three months post-procedure, and the percentage of total body weight loss (%TBWL) was calculated. Severe adverse events requiring hospital admission/procedure reversal were also recorded. We aim to see if one procedure is more efficient in providing weight loss in a short-term period.

Results

A total of 20 patients underwent ESG and 31 patients underwent IGB insertion. ESG patients showed a superior mean %TBWL at one-week post-procedure (%TBWL±SD = 4.87±1.88 vs 3.76±1.95). IGB patients showed a higher mean of %TBWL at one-month post-procedure (%TBWL±SD = 8.00±3.60 vs 7.25±3.29). Both procedures show similar %TBWL at three months post-procedure (%TBWL±SD ESG = 10.857±3.83 vs %TBWL ± SD IGB = 10.852±5.78).

Conclusions

We found that both the IGB insertion and ESG procedures result in clinically significant weight loss. However, the short-term weight loss between these two procedures is similar. Although similar, the number of adverse events in the IGB group is significantly higher than in the ESG group.

## Introduction

As a medical condition, obesity is a global public health concern that still has no satisfactory solution [1]. According to WHO, obesity is defined as abnormal or excessive fat accumulation that presents health risks [2]. Bariatric surgery is the gold standard for the management of moderate to severe obesity [3]. However, the percentage of patients eligible to undergo surgery is only 1-2% each year [3,4]. Obesity is the second leading cause of preventable death in the United States, currently outdone only by smoking [4]. The degrees of obesity are defined by body mass index (BMI = weight (kg)/height(m2), which correlates body weight with height [5].

The IGB is a space-occupying device that has been safely used to induce weight loss [6-8]. The fluid-filled balloons reduce available gastric volume and may delay gastric emptying [6,7]. Systematic review and meta-analysis reported that patients undergoing IGB therapy achieved 13.16% total body weight loss (TBWL) at six months [9]. A recent review demonstrated a 9.7 % TBWL at six months, with decreasing efficacy after six months [9-11].

Endoscopic sleeve gastroplasty (ESG) is a minimally invasive endoscopic procedure, where endoscopic full-thickness suturing is used to approximate the anterior and posterior walls of the stomach to achieve tubular reconfiguration and decrease gastric volume [9]. ESG and IGB are proven to be safe and efficient in producing weight loss. Choosing one procedure over another is based on patient preference after giving informed consent. We hypothesized that ESG would be superior and more efficient in providing weight loss with fewer postoperative complications compared with the IGB.

## Materials and methods

Patient population 

The method used in this research is a retrospective cohort using prospectively reviewed data from a total of 54 patients. Thirty-two patients underwent IGB placement, and 22 patients underwent ESG between June 2022 and June 2023 in a tertiary private hospital in Jakarta, Indonesia. The exclusion criteria are implant removal in the IGB group and normal BMI. Two patients with normal baseline BMI were excluded from the ESG group and one patient was excluded from the IGB group. From a total of 31 patients, five patients developed an intolerance to gastric balloons that required procedure reversal.

Before choosing the procedure, all patients had an initial consultation where they were provided with information about the weight loss program, where bariatric surgery is advised if other options failed to achieve sustainable weight loss. All procedures were self-paid, with the IGB program priced at approximately US$ 3,650 and the ESG program priced at approximately US$ 10,150. 

The participants provided their informed consent to participate in this study. The Strengthening the Reporting of Observational studies in Epidemiology (STROBE) guidelines were used to ensure the reporting of this retrospective cohort study.

Pre and post-procedural care 

All procedures were performed by a team of surgeons experienced in endoscopic suturing and intragastric balloon placement. Preoperative medication for the IGB placement patients' group is omeprazole for 10 days. The post-OP medication for IGB group patients is omeprazole for as long as the IGB is inside the patient’s stomach. Postoperative, the diet is restricted to a liquid diet for two weeks post-procedure after which it is transitioned to a soft diet and a solid diet if it’s tolerated individually.

Intragastric balloon (IGB) insertion 

Indications for IGB placement were a body mass index (BMI) > 27 kg/m^2^ in patients who had previously been unsuccessful in losing weight through diet, and/or exercise, and/or medications. Contraindications are patients' history of gastroesophageal surgery and active anticoagulation.

The balloon used was Orbera. The balloons were inserted endoscopically as per the manufacturer’s recommendations and filled with 500-550 cc of normal saline infused with methylene blue to detect leakage. All balloon insertions were performed with patients under general anesthesia in the operating room.

Endoscopic sleeve gastroplasty (ESG)

Indications for ESG were BMI >30 kg/m^2^ in patients who had been unsuccessful in losing weight despite undergoing diet, exercise, and/or medications. Contraindications for ESG are a history of gastric cancer, active Helicobacter (H.) pylori infection, active gastric ulcer, gastric intestinal metaplasia, and organ failure. All ESG procedures were performed on patients under general anesthesia in the operating room.

Outcomes

Patients’ data collected were age, sex, initial weight, height, and initial BMI. The patients were contacted by a clinical nurse via phone in the first week post-procedure and one month and three months post-procedure. The primary outcome was TBWL and %TBWL at each time point and adverse events were recorded at follow-up. Adverse events were recorded only if the patient required admission to the hospital. Nausea, vomiting, and abdominal discomfort were expected post-procedure and were not recorded.

Statistical analysis

Descriptive statistics were calculated for all demographic and clinical variables and presented as mean or standard deviation (SD). Statistical analysis was done using an independent-samples t-test for comparing the means of TBWL and %TBWL in each period post-operative (one week, one month, and three months post-procedure). All statistical analysis was conducted using SPSS version 23 (IBM Corp. Released 2015. IBM SPSS Statistics for Windows, Version 23.0. Armonk, NY: IBM Corp).

Ethical approval

The participants provided their informed consent to participate in this study. The Sumber Waras Hospital ethical committee approved this study with the registered number 12/RSSW/KoM.EP/EC/X/2023.

## Results

**Figure 1:**
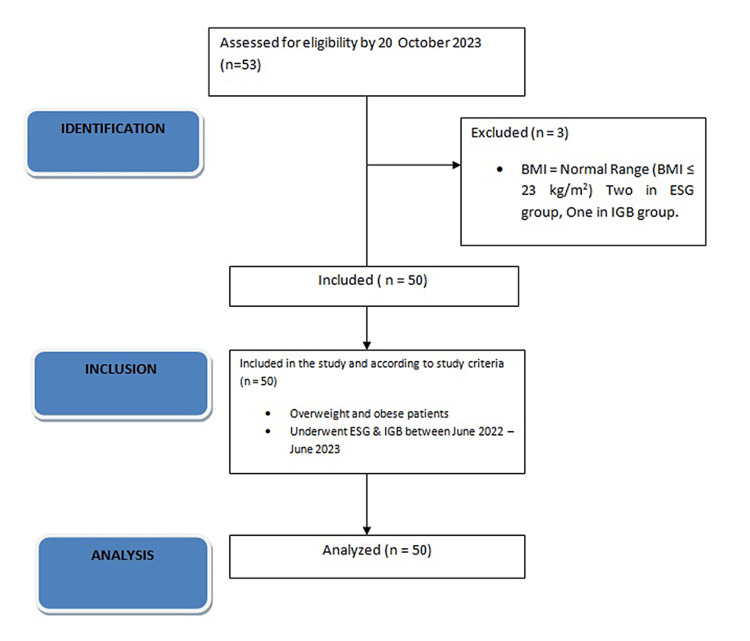
STROBE flowchart of the research process BMI, body mass index; ESG, endoscopic sleeve gastroplasty; IGB, intragastric balloon insertion; STROBE: Strengthening the Reporting of Observational studies in Epidemiology

From a total of 54 patients, two patients with normal BMI were excluded from the ESG group, and one patient was excluded from the IGB group. The group was divided into those who underwent endoscopic sleeve gastroplasty (ESG) and those who underwent intragastric balloon insertion (IGB). A total of 20 patients underwent ESG, and a total of 31 patients received IGB. The baseline characteristics of the two groups are further described in Table 1. The flowchart describing the research process is shown in Figure 1.

**Table 1:** Baseline characteristics of 51 patients who underwent ESG and IGB ESG, endoscopic sleeve gastroplasty; IGB, intragastric balloon

	ESG	IGB
Age, mean ±SD, years	40.05±10.54	38.67±8.41
Sex, female, n (%)	17 (85.0%)	24 (77.4%)
BMI, mean±SD, kg/m^2^	31.12±5.88	31.16±4.39

The mean age is comparable between the ESG group and the IGB group, with the IGB group having a lower mean age (40.05 vs 38.67). The number of females in the two groups was significantly higher than the males (ESG: 85% and IGB: 77.4%). The baseline BMI between the two groups is also similar (ESG: 31.12 kg/m^2^, IGB:31.16 kg/m^2^). Patients’ BMI ranges from 24.54 kg/m^2^ to 53.97 kg/m^2^ in the ESG group and ranges from 24.05 kg/m^2^ to 46.88 kg/m^2^ in the IGB group. The comparison of weight loss between these two procedures is further shown in Table 2 and Table 3.

**Table 2:** Percentage of total body weight loss of patients followed up at one week, one month, and three months post-procedure *P-value (𝑝) derived from the student's t-test. ESG, endoscopic sleeve gastroplasty; IGB, intragastric balloon; TBWL, total body weight loss

Post-procedure period	Procedure	Number of patients followed up, n (%)	Mean of %TBWL±SD	P-value (𝑝)
1 week	ESG	20 (100)	4.87±1.88	0.053*
	IGB	31 (100)	3.76±1.95	0.052
1 month	ESG	18 (90.00)	7.25±3.29	0.481
	IGB	27 (87.09)	8.00±3.60	0.473
3 months	ESG	15 (75.00)	10.857±3.83	0.998
	IGB	21 (67.74)	10.852±5.78	0.998

**Table 3:** Total body weight loss (kg) of patients followed up at one week, one month, and three months post-procedure *P-value (𝑝) derived from students t-test. ESG, endoscopic sleeve gastroplasty; IGB, intragastric balloon; TBWL, total body weight loss

Post-procedure period	Procedure	Number of patients followed-up, n (%)	Mean of TBWL, kg (SD)	P-value (𝑝)
1 week	ESG	20 (100)	3.89±1.56	0.161*
	IGB	31 (100)	3.13±2.04	0.139
1 month	ESG	18 (90.0)	5.94±2.75	0.461
	IGB	27 (87.0)	6.76±4.09	0.426
3 months	ESG	15 (75.0)	8.68±3.56	0.731
	IGB	21 (67.7)	9.29±6.05	0.709

The ESG group has a higher mean of %TBWL and TBWL (kg) in one-week post-procedure (%TBWL±SD = 4.87±1.88% vs. 3.76±1.95; P = 0.053, TBWL (kg) = 3.89 (1.56) vs 3.13 (2.04); P=0.16). Although it has a lower one-week weight loss, the IGB group shows higher means of %TBWL in one month (%TBWL (SD) = 8.00% (3.60%) vs 7.25% (3.29%); P = 0.91). The IGB group also yields a higher mean of TBWL (kg) in one month (TBWL (kg) (SD) = 6.76 (4.09) vs 5.94 (2.75); P = 0.46) and TBWL (kg) in 3 months (TBWL (kg) (SD) = 9.29 (6.05) vs 8.68 (3.56); P = 0.73) post-procedure. Surprisingly, the ESG and IGB groups show almost similar means of %TBWL in three months post-procedure (%TBWL (SD) = 10.857 vs 10.852 (5.78); P = 0.998).

Comparing the complication rates, there was a higher complication in the IGB group that required early removal (IGB: 16.1%, ESG: 0%).

## Discussion

One week after the procedure, the ESG group showed higher means of %TBWL than the IGB group (4.87% vs 3.76%). Although it averaged higher, the differences between the two groups were not significant statistically ( P>0.05). In the one-month post-procedure follow-up, the IGB group provided a higher mean of percentage weight loss compared to the ESG group (8.08% vs 7.25%). Although higher, the difference was not statistically more significant than the ESG group. This difference is, however, almost non-existent at the three-month post-procedure follow-up, where the IGB group showed a slightly lower %TBWL than the ESG group (%TBWL = 10.852% vs 10.857%). Comparing our results to the previous study, there was a notable difference in %TBWL at the one-month and three-month post-procedure follow-up in the ESG group [12]. A 2018 study by Sartoretto et al. documented 8.8% (2.5%) %TBWL in the one-month post-procedure follow-up and up to 12% (1.3%) in %TBWL at three months post-procedure in ESG patients [13]. A previous study by Fayed et al. in 2019 also showed a higher %TBWL at one-month and three-months post-procedure (9.9% (2.4%) and 14.3% (4.6%)) [12]. In contrast, the IGB group showed a higher %TBWL at one month (8.0% (3.6%) vs 6.6% (2.6%)) but a lower %TBWL at three months post-procedure (10.85% (5.78) vs 11.1% (4.4%)) than the Fayad et al. study [12].

We believe that the difference in %TBWL in our study compared with previous ones may be attributed to the significant gap in average BMI. A multi-center study by Lopez-Nava et al. in 2019 stated that a higher initial BMI predicted a higher %TBWL in one year [14]. The ESG group of a previous study by Fayed et al. had a mean BMI of 41.5 (8.5) vs 31.12 (5.88) in our group. While the IGB group base BMI is not similar between the two studies, the gap is not too significant (31.16 vs 34.5), hence the result is more similar in the IGB group.

In the three-month post-procedure follow-up, the %TBWL between the ESG and IGB groups became similar, with no group superior to the other in weight loss efficacy. This result supports a previous study by Lopez-Nava et al. in which weight loss at one year was not dependent on the type of procedure. However, in the same study, Lopez-Nava et al. state that follow-up attendance is a determining factor in providing weight loss. Frequent interaction with the multi-disciplinary team (MDT) at follow-up might have provided an opportunity to identify "at-risk-of-failure" patients and intervene at an early stage. Besides, the psychological counseling and motivation of early responders may have promoted sustained weight loss at one year [14,15].

While the efficacy between the two procedures is similar in short-term weight loss, the IGB group in our study has a higher percentage of intolerance of balloon insertion in 5 of 31 patients, a total of 16.1% compared with 0% from the ESG group. This rate of complication in the IGB group is also similar to the previous study by Fayed et al. (17%) [16]. Another previous study shows a lower percentage of early removals (7%, 9%, and 2.6%) [15]. Even though it is rare, complications for ESG are more serious and concerning (perigastric fluid collection, blood loss) [16,17]. In a previous study by Asokkumar et al. concerning the safety and early efficacy of ESG in Singapore, there is also no major complication rate recorded [18]. Multiple studies involving the Western population have established the safety and efficacy of ESG. Hedjoudje et al., in a meta-analysis involving 1772 ESG patients, showed the rate of adverse events to be 2.2% [16].

Although retrospective, our single-centered study had a similar care program and surgery team. There is a similar preoperative preparation and also similar post-procedural care. Patients in both groups also have access to the same group of dieticians who guide patients’ diet post-procedure. However, our study is also limited by the number of patients available, further studies may be needed with a higher number of patient population. We acknowledge the limitation of loss of follow-up in our patient population, which may be attributed to its retrospective model. Some loss of follow-up is also caused by patients who live in distant regions.

## Conclusions

In conclusion, our study shows that ESG and IGB have similar efficacy in providing weight loss in a short-term period. Significant weight loss occurs even in a three-month period post-procedure without major morbidity or mortality. Nausea and abdominal pain are a common post-procedural complication, but the IGB group shows a notable rate of removal. As a pilot study in our center, further prospective study, especially with multicenter backgrounds with larger patient populations, is needed with long-term follow-up comparing these procedures with the same patient background.
